# Diagnosis and prevalence of Congenital Adrenal Hyperplasia (CAH) in Austrian children screened or not screened for CAH

**DOI:** 10.1186/1687-9856-2013-S1-P111

**Published:** 2013-10-03

**Authors:** Katharina Luxenberger, David Kasper, Elke Fröhlich-Reiterer, Elisabeth Suppan, Gudrun Weinhandl, Martin Borkenstein

**Affiliations:** 1 Departments of Pediatrics and Adolescence Medicine, Medical Universities Graz, Graz, Austria; 2 Departments of Pediatrics and Adolescence Medicine, Medical Universities Vienna, Vienna, Austria

## 

Prevalence of Congenital Adrenal Hyperplasia (CAH) is not exactly known in the Austria; a number of patients with CAH might not be diagnosed, especially males. CAH is in about 95 % of the cases due to a defect in the 21-hydroxylation (‘classical CAH’). Newborn screening for CAH, based on the measurement of 17α-hydroxyprogesterone (17-OHP) was shown to be efficient for diagnosis, and is part of the newborn screening programme in Austria since April 2001.

In our study we compared 2 groups of children:

Group A, children born in Styria (a province of Austria), 1992 – 2001, n = 119.001, m 61.256, and f 57.745;

Group B, children born in Styria 2002 – 2011, n = 103.228, m 52.722, and f 50.506

In group A (patients not screened), CAH was diagnosed in 8 children (m 4; f 4); 4 of them with simple virilising (SV) 21-OH deficiency (m 3; f 1) and 4 with salt wasting (SV) 21-OH deficiency (m 1; f 3).

In group B, 98,7 % of all newborns born in Styria could be screened by measuring 17-OHP in a dried blood spot on filter paper. Recall rate was 0,578 %. CAH was diagnosed in 10 children (m 3: f 7). 8 of them with SW (m 2; f 6), and 2 with 11β-hydroxylase deficiency (m 1; f 1).

Whereas group A displayed the expected Mendelian sex ratio, group B showed a strong female predominance (m 3; f 7)

Prevalence of CAH was 1:14.875 newborns in group A (not screened). In group B (newborns screened) prevalence was 1:10.132.

If one exclude the 2 patients with 11ß-hydroxylase deficiency from group B, prevalence of ‘classical CAH’ was 1:14.875 in group A, compared to 1:12.903 in group B.

These data show that newborn screening for CAH seems to increase the rate of detection of CAH.

